# Supplementary material for: Effects of eicosapentaneoic acid on innate immune responses in an Atlantic salmon kidney cell line in vitro
Source: PLoS One. 2024 May 28;19(5):e0302286. doi: 10.1371/journal.pone.0302286 (PMC11132502; doi:10.1371/journal.pone.0302286)
Supplement: S1 File — (PDF) [file pone.0302286.s001.pdf]

Effects of eicosapentaneoic acid on innate immune responses in an Atlantic salmon kidney cell line in vitro

Tor Gjøen, Bente Ruyter and Tone Kari Knutsdatter Østbye

23 February, 2024

This supplementary file provides sample information and exploratory plots for the manuscript "Effects of EPA on poly I:C-stimulated genes in Atlantic salmon (Salmo salar) head kidney cells". It shows the RNAseq mapping summary, rnaseq count data distributions, replicate correlations and sample clustering. The analysis have been performed using the R statistical programming language and R BioConductor packages (a complete list is given in the bottom of the document (Session info)). This supplementary file was generated using the knitr R package under Windows 10.

S1 Table

Sample_no	Sample_name	EPA_uM	polyIC	Total_reads	Overall.alignment_rate
1	Control_1	0	0	20,326,182	82.4 %
2	Control_2	0	0	18,192,144	78.1 %
3	Control_EPA_1	200	0	18,499,991	83.8 %
4	Control_EPA_2	200	0	19,851,170	81.7 %
5	Control_pIC_1	0	30	19,036,302	82.2 %
6	Control_pIC_2	0	30	16,889,434	82.0 %
7	EPA_25uM_1	25	30	20,040,044	82.1 %
8	EPA_25uM_2	25	30	15,294,322	81.5 %
9	EPA_50uM_1	50	30	22,865,954	81.9 %
10	EPA_50uM_2	50	30	25,907,567	82.9 %
11	EPA_100uM_1	100	30	19,214,264	84.6 %
12	EPA_100uM_2	100	30	20,933,753	83.5 %
13	EPA_200uM_1	200	30	22,786,140	83.7 %
14	EPA_200uM_2	200	30	20,209,166	83.5 %
15	Control_3	0	0	16,006,100	80.6 %
16	Control_4	0	0	19,041,384	84.1 %

Sample_no	Sample_name	EPA_uM	polyIC	Total_reads	Overall.alignment_rate
17	Control_EPA_3	200	0	17,160,656	82.8 %
18	Control_EPA_4	200	0	19,253,064	84.5 %
19	Control_pIC_3	0	30	24,677,862	83.7 %
20	Control_pIC_4	0	30	19,579,332	83.7 %
21	EPA_25uM_3	25	30	23,078,389	83.8 %
22	EPA_25uM_4	25	30	17,900,356	84.9 %
23	EPA_50uM_3	50	30	19,795,914	83.9 %
24	EPA_50uM_4	50	30	19,341,202	82.2 %
25	EPA_100uM_3	100	30	22,033,381	82.3 %
26	EPA_100uM_4	100	30	21,046,322	83.2 %
27	EPA_200uM_3	200	30	22,098,566	84.3 %
28	EPA_200uM_4	200	30	20,673,260	84.6 %
29	Control_5	0	0	19,536,871	83.9 %
30	Control_6	0	0	16,967,234	85.5 %
31	Control_EPA_5	200	0	18,704,934	77.9 %
32	Control_EPA_6	200	0	19,888,471	84.6 %
33	Control_pIC_5	0	30	17,342,620	85.0 %
34	Control_pIC_6	0	30	17,510,227	81.7 %
35	EPA_25uM_5	25	30	19,267,147	83.2 %
36	EPA_25uM_6	25	30	20,201,062	85.4 %
37	EPA_50uM_5	50	30	18,992,674	84.1 %
38	EPA_50uM_6	50	30	17,547,667	83.9 %
39	EPA_100uM_5	100	30	19,012,456	83.8 %
40	EPA_100uM_6	100	30	18,206,228	82.9 %
41	EPA_200uM_5	200	30	17,174,476	83.2 %
42	EPA_200uM_6	200	30	19,727,336	84.3 %

S1 Fig

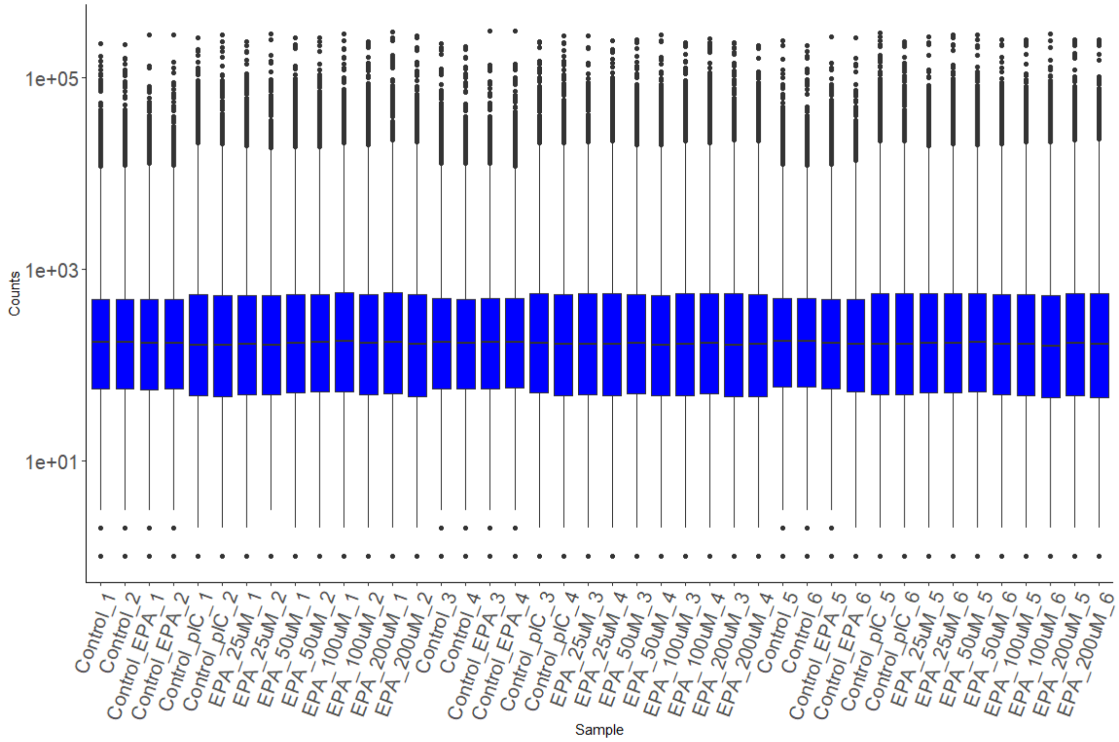

Boxplot display of the distribution of counts in each sample. Mean and variance were comparable in all samples.

S2 Fig

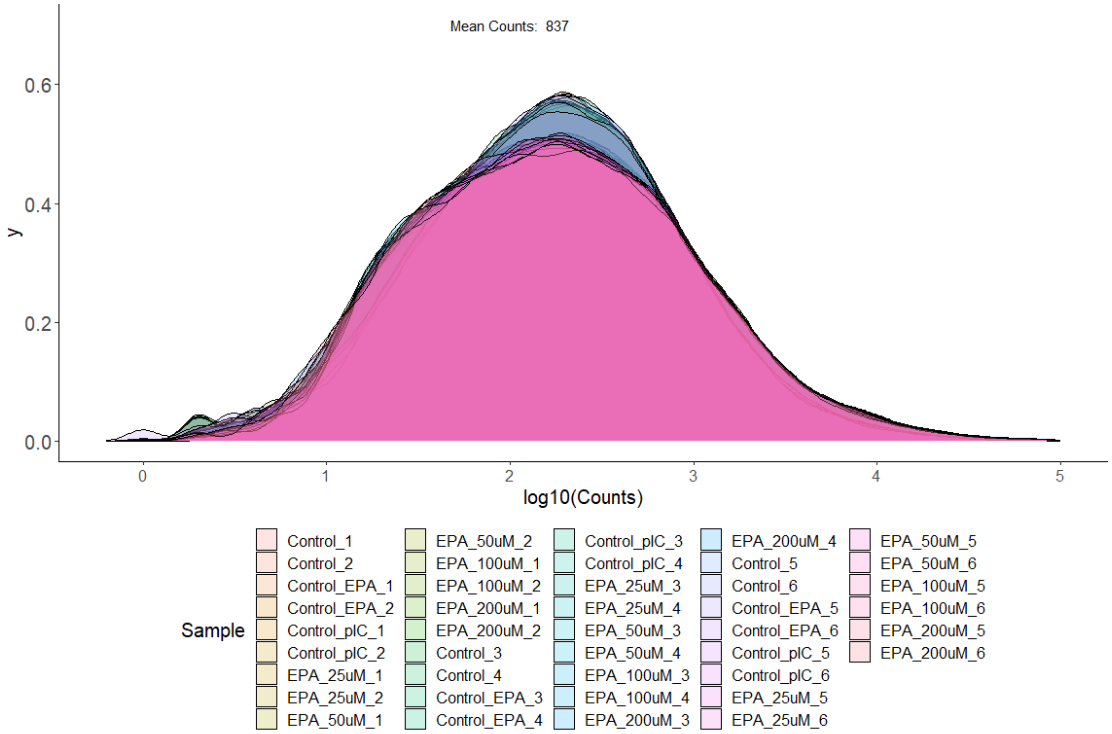

Density plot comparing the distribution of log10 raw counts in each sample

S3 Fig

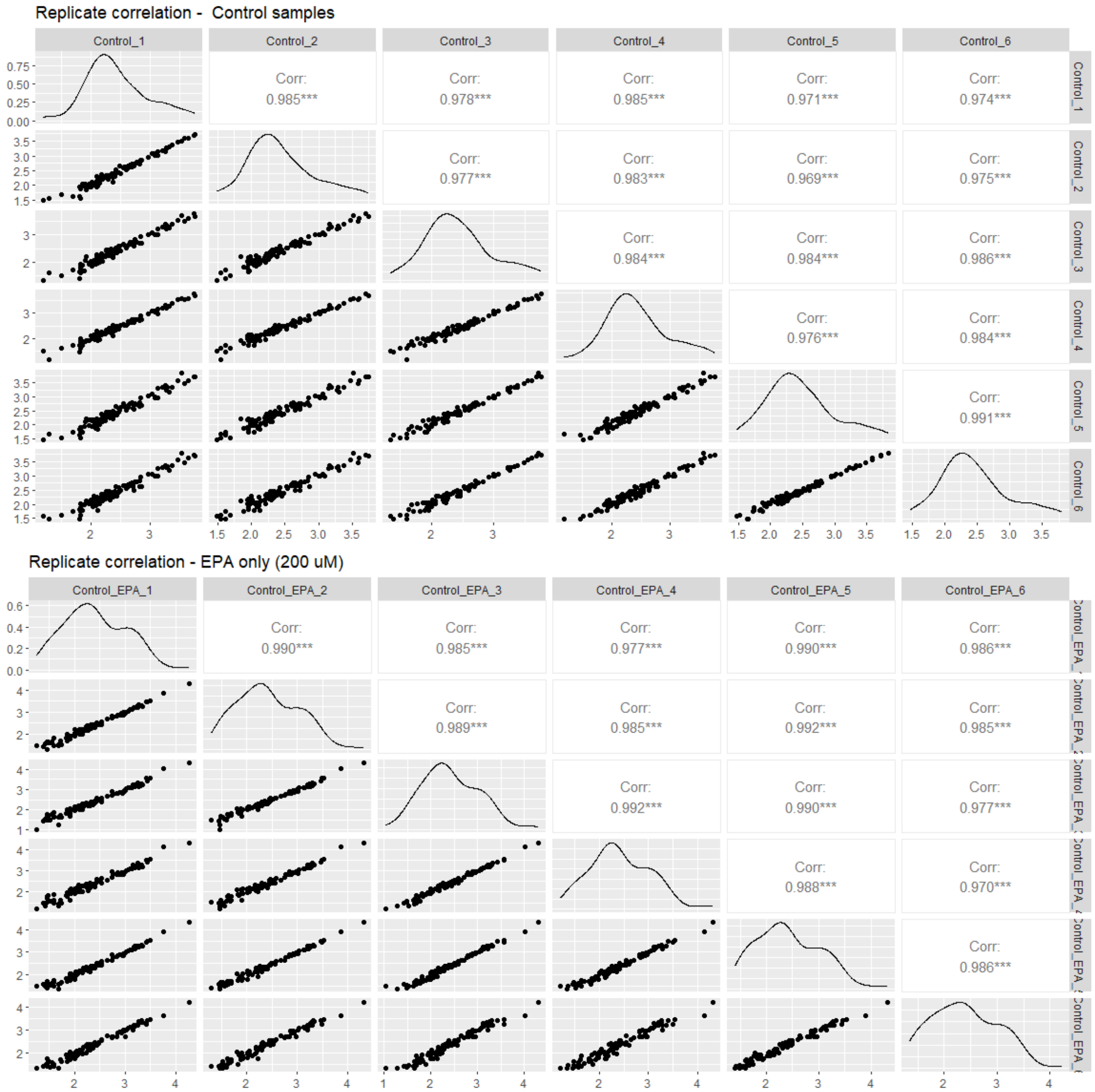

Replicate correlation - Control samples
Control_1
Control_2
Control_3
Control_4
Control_5
Control_6
Corr: 0.985***
Corr: 0.978***
Corr: 0.985***
Corr: 0.971***
Corr: 0.974***
Corr: 0.977***
Corr: 0.983***
Corr: 0.969***
Corr: 0.975***
Corr: 0.984***
Corr: 0.984***
Corr: 0.986***
Corr: 0.976***
Corr: 0.984***
Corr: 0.991***
Replicate correlation - EPA only (200 uM)
Control_EPA_1
Control_EPA_2
Control_EPA_3
Control_EPA_4
Control_EPA_5
Control_EPA_6
Corr: 0.990***
Corr: 0.985***
Corr: 0.977***
Corr: 0.990***
Corr: 0.986***
Corr: 0.989***
Corr: 0.985***
Corr: 0.992***
Corr: 0.985***
Corr: 0.992***
Corr: 0.990***
Corr: 0.977***
Corr: 0.988***
Corr: 0.970***
Corr: 0.986***

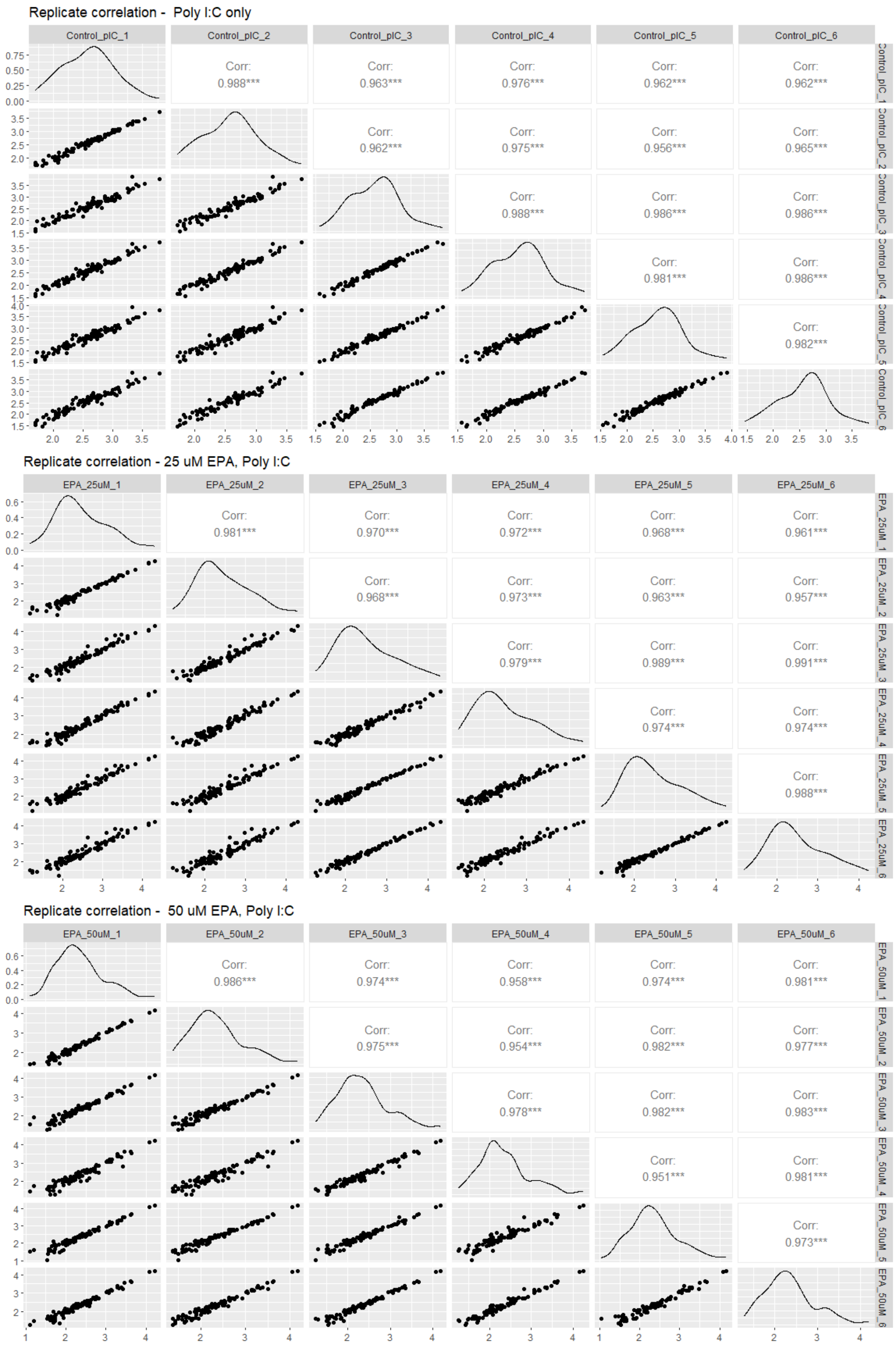

Replicate correlation - Poly I:C only
Control_pIC_1
Control_pIC_2
Control_pIC_3
Control_pIC_4
Control_pIC_5
Control_pIC_6
Corr: 0.988***
Corr: 0.963***
Corr: 0.976***
Corr: 0.962***
Corr: 0.962***
Corr: 0.962***
Corr: 0.975***
Corr: 0.956***
Corr: 0.965***
Corr: 0.988***
Corr: 0.986***
Corr: 0.986***
Corr: 0.981***
Corr: 0.986***
Corr: 0.982***
Replicate correlation - 25 uM EPA, Poly I:C
EPA_25uM_1
EPA_25uM_2
EPA_25uM_3
EPA_25uM_4
EPA_25uM_5
EPA_25uM_6
Corr: 0.981***
Corr: 0.970***
Corr: 0.972***
Corr: 0.968***
Corr: 0.961***
Corr: 0.968***
Corr: 0.973***
Corr: 0.963***
Corr: 0.957***
Corr: 0.979***
Corr: 0.989***
Corr: 0.991***
Corr: 0.974***
Corr: 0.974***
Corr: 0.988***
Replicate correlation - 50 uM EPA, Poly I:C
EPA_50uM_1
EPA_50uM_2
EPA_50uM_3
EPA_50uM_4
EPA_50uM_5
EPA_50uM_6
Corr: 0.986***
Corr: 0.974***
Corr: 0.958***
Corr: 0.974***
Corr: 0.981***
Corr: 0.975***
Corr: 0.954***
Corr: 0.982***
Corr: 0.977***
Corr: 0.978***
Corr: 0.982***
Corr: 0.983***
Corr: 0.951***
Corr: 0.981***
Corr: 0.973***

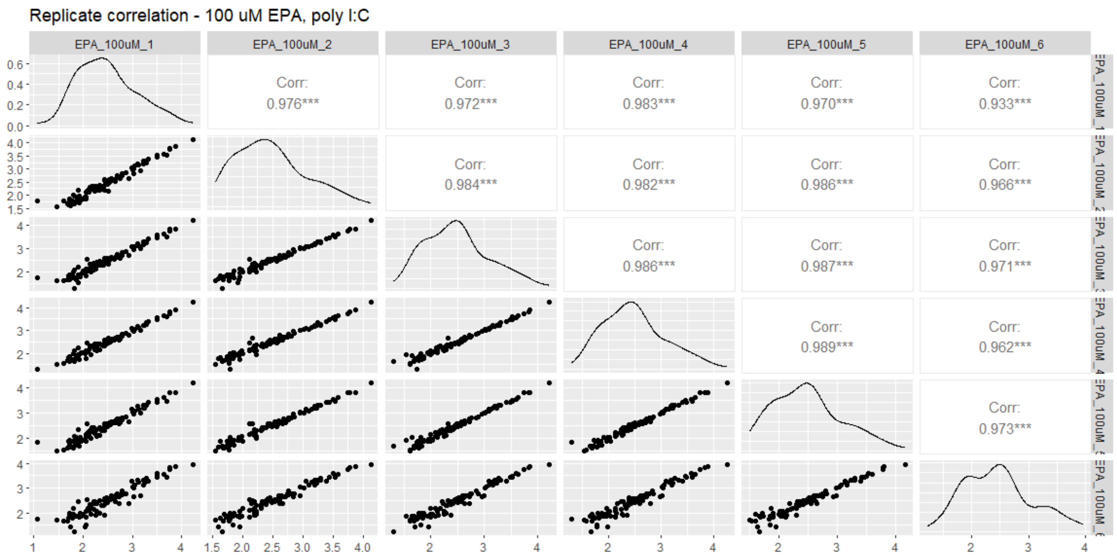

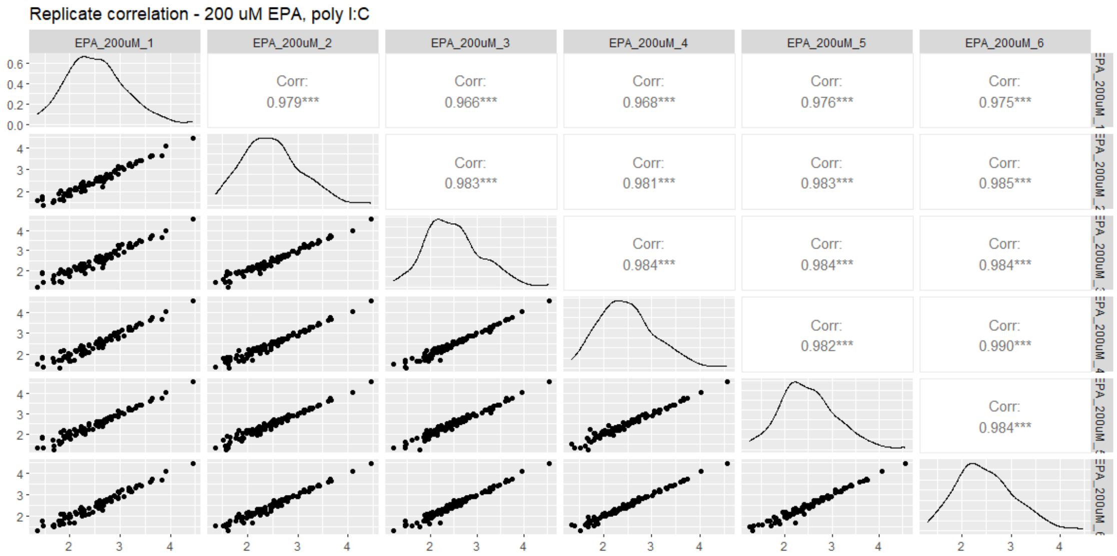

Plots showing sample data correlation between 100 randomly selected genes with at least 10 counts in all samples for each experimental group. The panels along the diagonal display the density distribution of counts in the selected samples and the right side of the panel shows the Pearson correlation coefficient for each sample pair.

S4 Fig

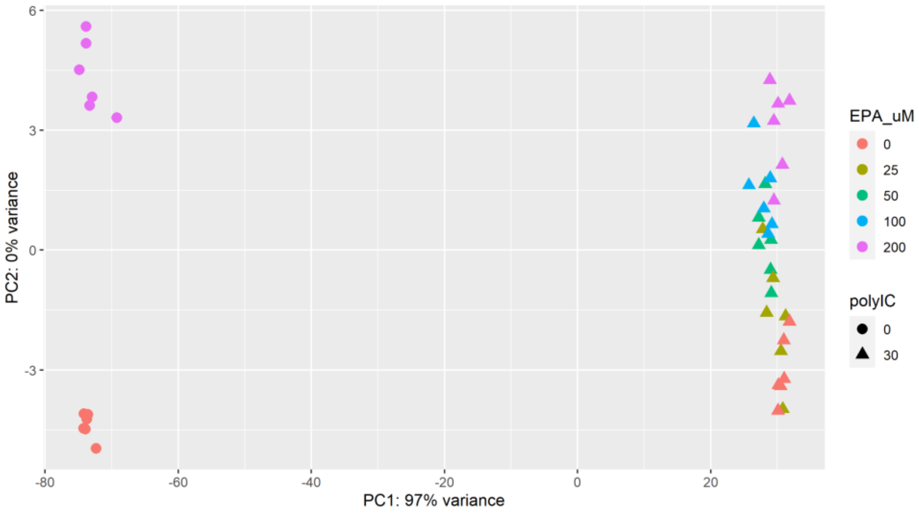

Principal component analysis of vst transformed gene expression values (50 most variable genes) shows that most of the variation in gene expression could be assigned to poly I:C. The level of EPA also contributes to variation in the dataset.”

S5 Fig

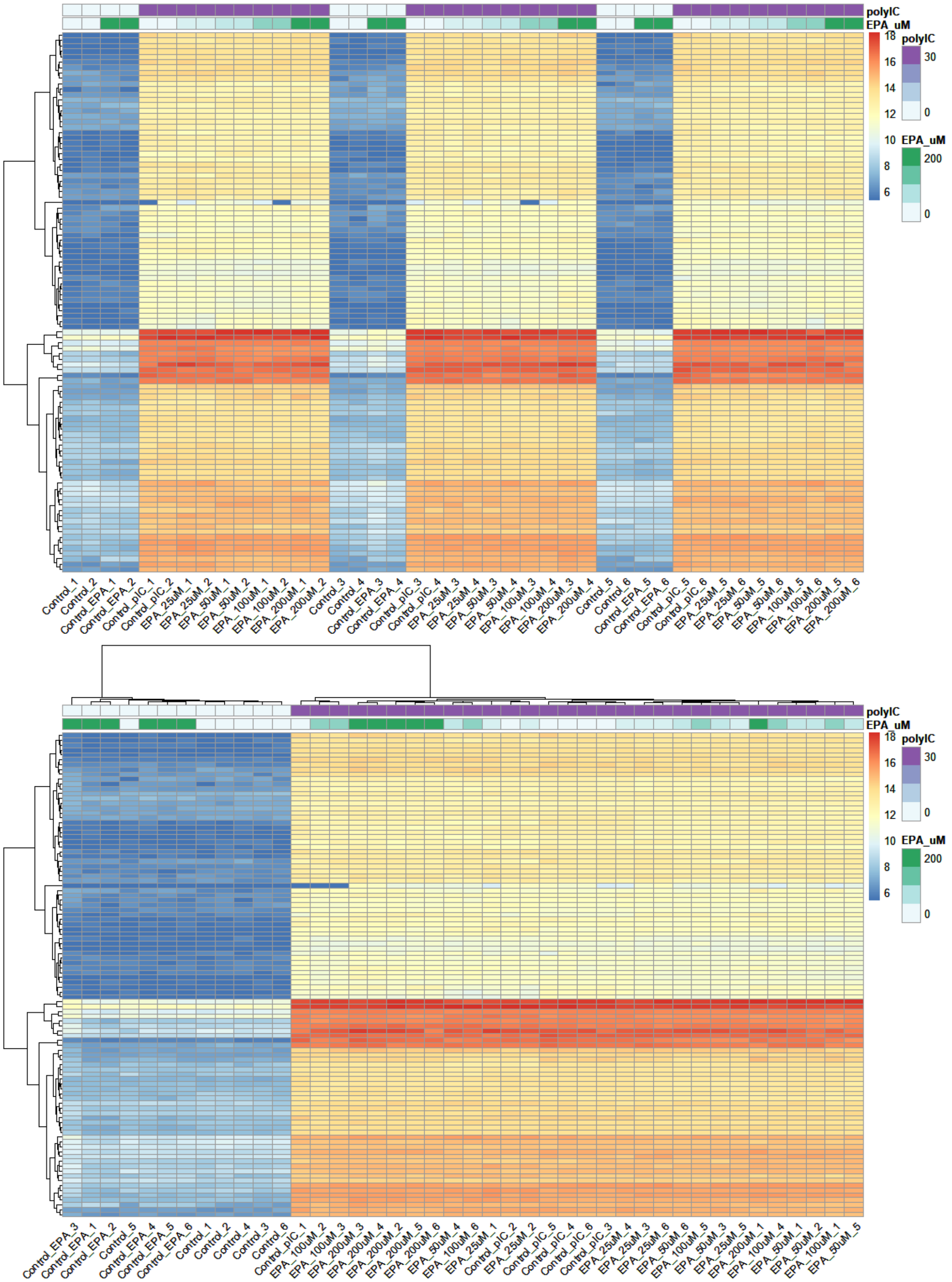

Hierarchical clustering heatmap (euclidian distance) of vst transformed counts from the 100 most variable genes. Upper panel shows clustering of genes into mainly two groups

(sensitive to poly (I:C)). Lower panel also clusters samples into mainly two groups affected by poly (I:C) or not.

S6 Fig

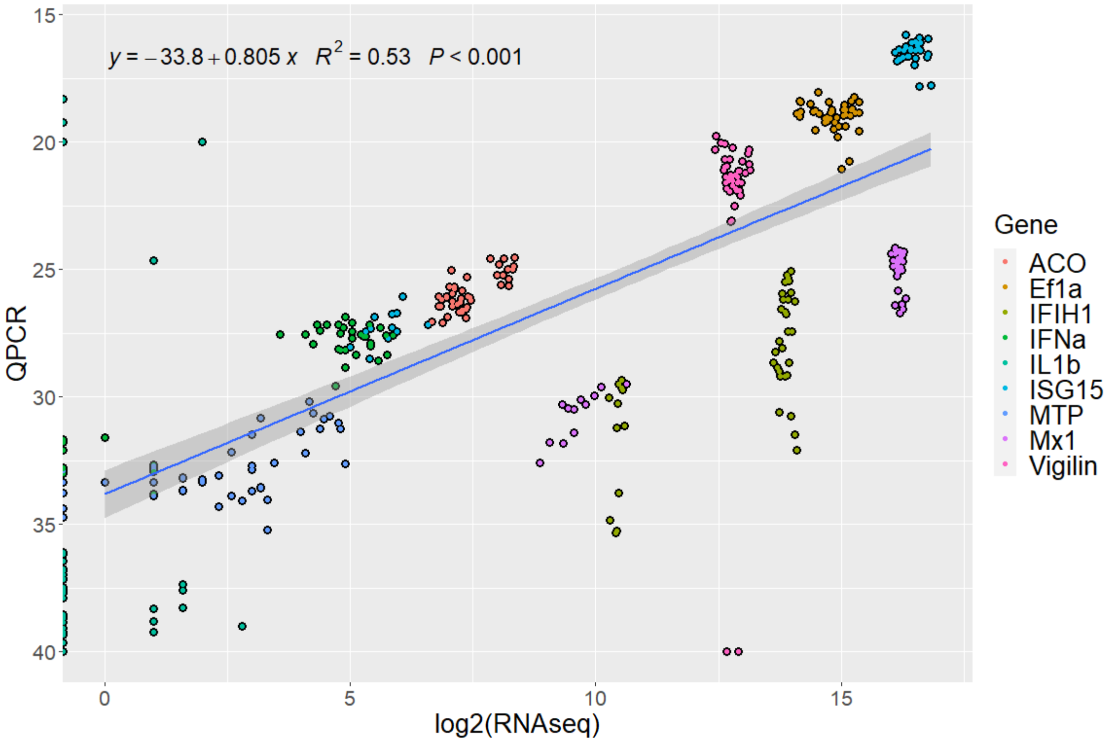

Correlation between QPCR and RNAseq analyses of Atlantic salmon head kidney cells incubated with various levels of EPA before stimulation with poly (I:C).

S7 Fig

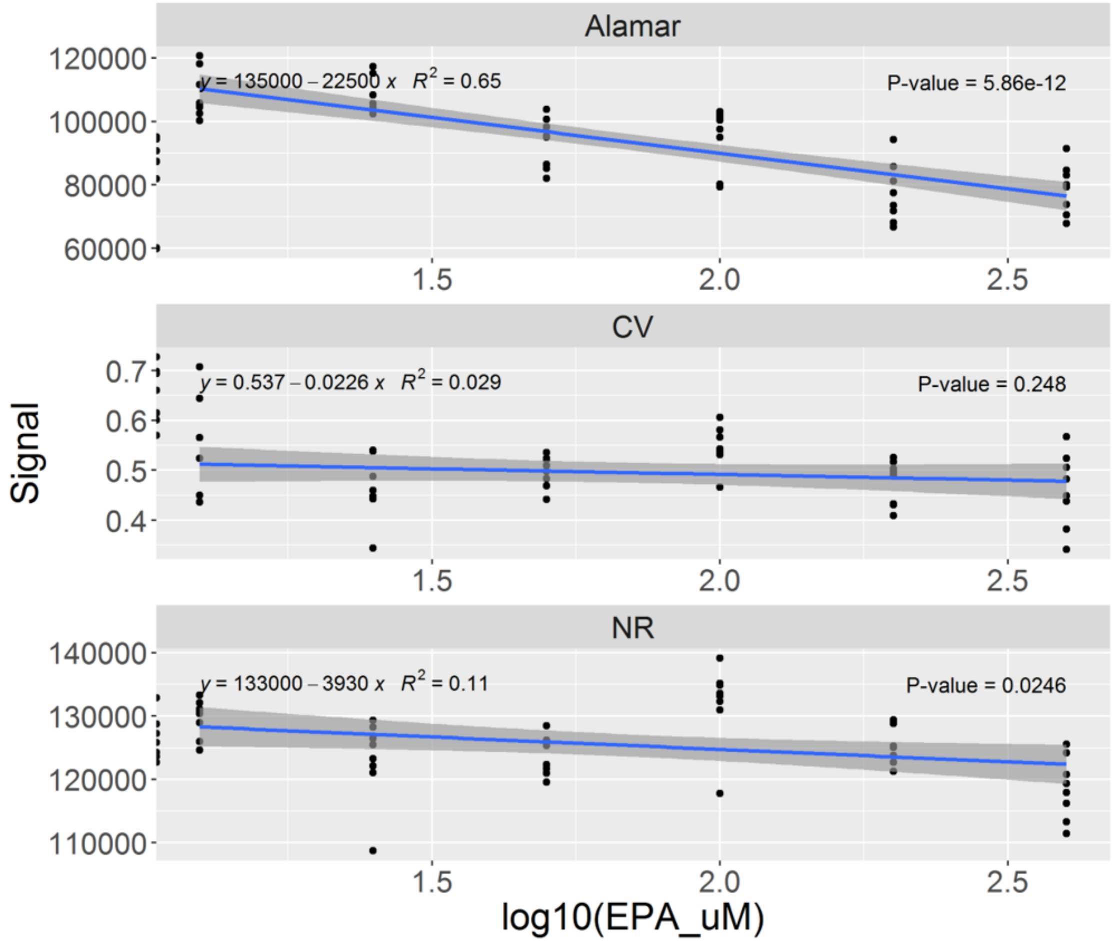

Effect of increasing amounts of EPA (up to 400 uM) in the cell culture medium of ASK cells on cell viability. Viability was analysed by Alamar blue assay, crystal violet staining of adherent cells (CV) and uptake of neutral red (NR)

S8 Fig

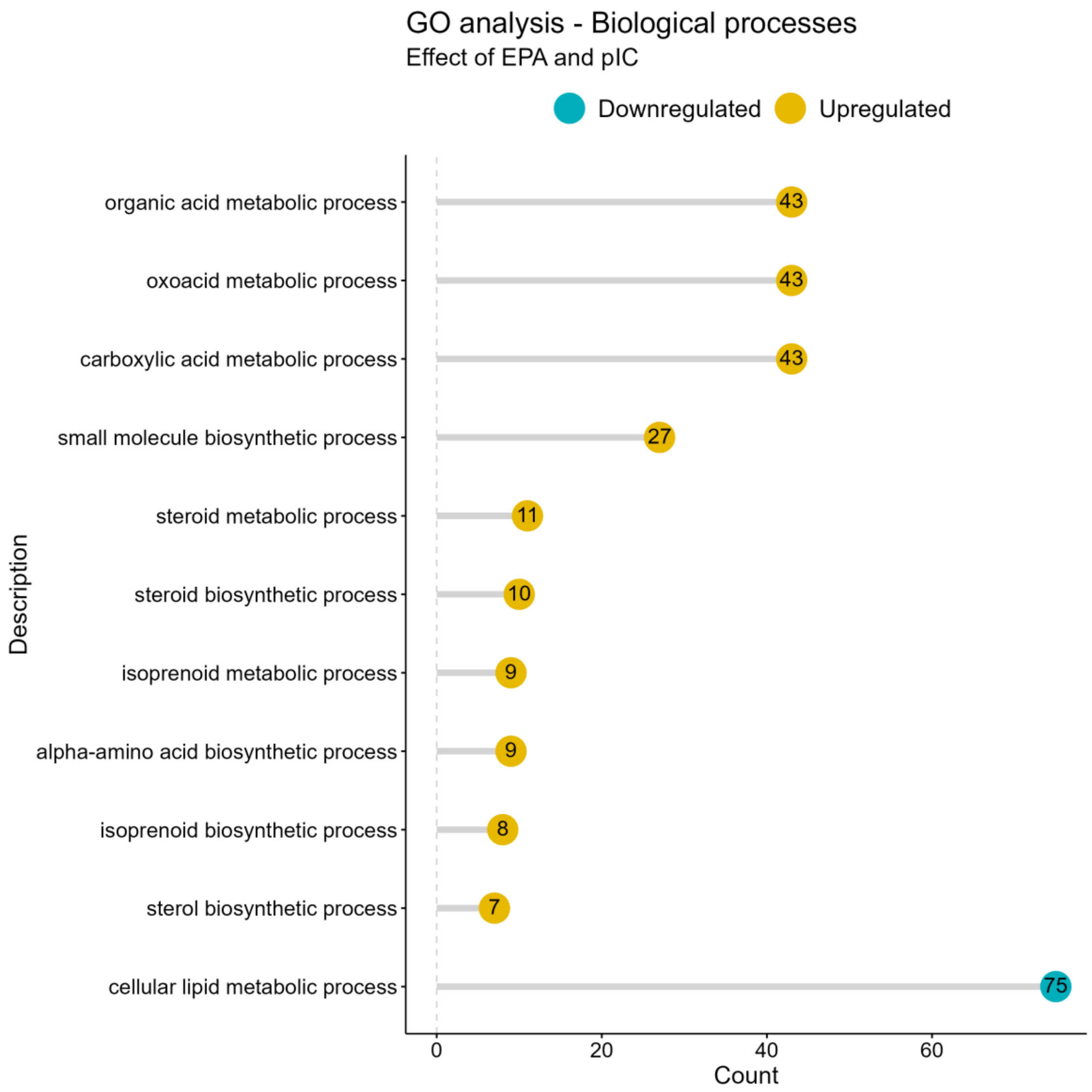

Enriched biological processes in ASK cells incubated with increasing levels of EPA and stimulated with poly I:C (30 ug/ml, 24 h).

S9 Fig

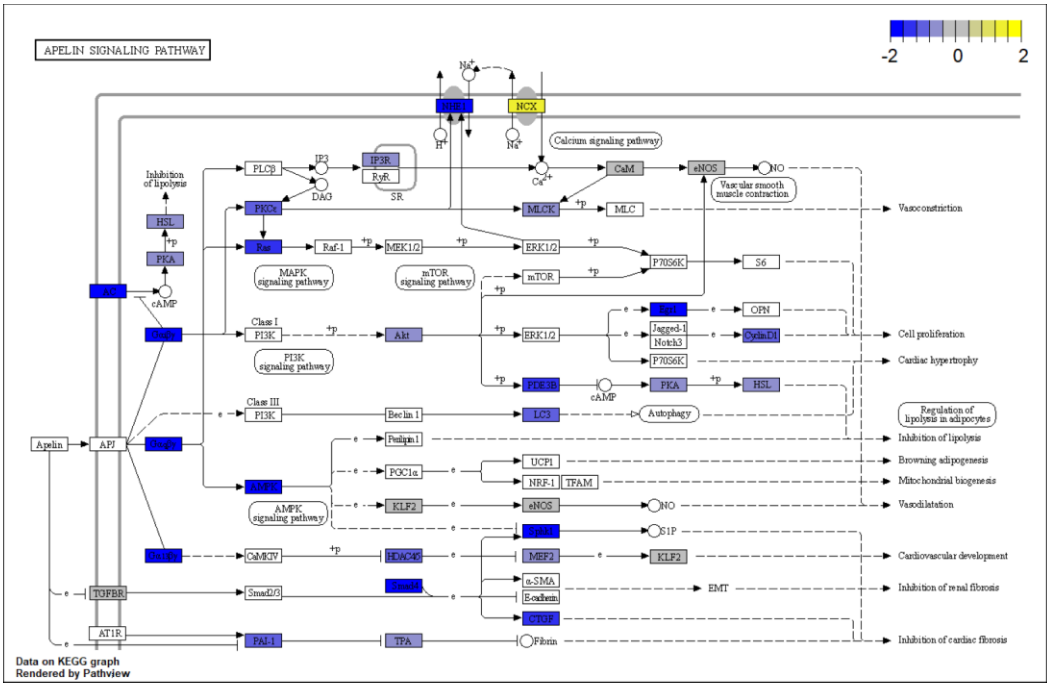

Effect of EPA (average log2 fold change) on ASK cell transcripts after poly I:C stimulation (30 ug/ml, 24 h) in the apelin signalling pathway.

S10 Fig

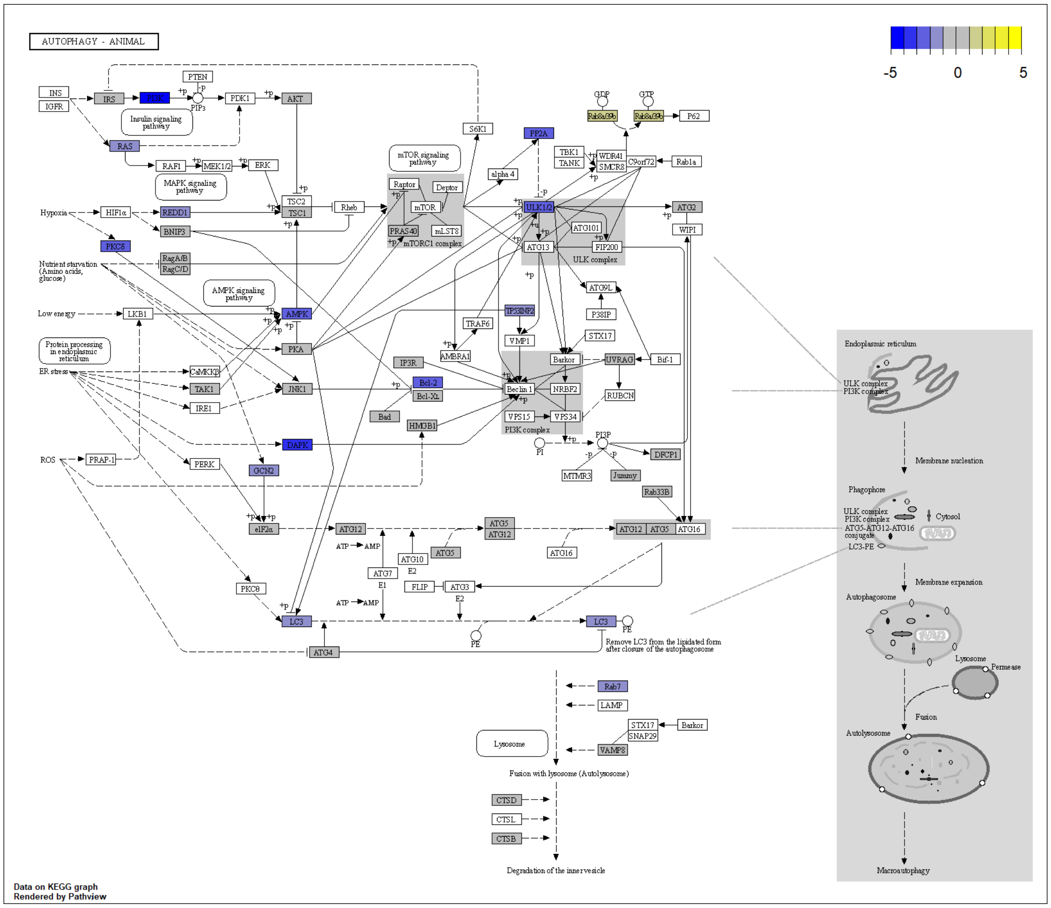

Effect of EPA (average log2 fold change) on ASK cell transcripts after poly I:C stimulation (30 ug/ml, 24 h) in the autophagy pathway.

Session information with list of R packages

```
## R version 4.3.1 (2023-06-16 ucrt)
## Platform: x86_64-w64-mingw32/x64 (64-bit)
## Running under: Windows 10 x64 (build 19045)
##
## Matrix products: default
##
##
## locale:
## [1] LC_COLLATE=English_United States.utf8
## [2] LC_CTYPE=English_United States.utf8
## [3] LC_MONETARY=English_United States.utf8
## [4] LC_NUMERIC=C
## [5] LC_TIME=English_United States.utf8
##
## time zone: Europe/Oslo
## tzcode source: internal
##
```

```
## attached base packages:
## [1] stats4    stats     graphics  grDevices utils     datasets  methods  
## [8] base     
## 
## other attached packages:
##  [1] readxl_1.4.3                ggpmisc_0.5.5              
##  [3] ggpp_0.5.6                  ggsignif_0.6.4             
##  [5] ggpubr_0.6.0                data.table_1.15.0          
##  [7] polynom_1.4-1               pheatmap_1.0.12            
##  [9] readr_2.1.5                 png_0.1-8                  
## [11] flextable_0.9.4             pander_0.6.5               
## [13] drc_3.0-1                   MASS_7.3-60.0.1            
## [15] DESeq2_1.42.0               SummarizedExperiment_1.32.0
## [17] Biobase_2.62.0              MatrixGenerics_1.14.0      
## [19] matrixStats_1.2.0           GenomicRanges_1.54.1       
## [21] GenomeInfoDb_1.38.5         IRanges_2.36.0             
## [23] S4Vectors_0.40.2            BiocGenerics_0.48.1        
## [25] genefilter_1.84.0           factoextra_1.0.7           
## [27] GGally_2.2.0                dplyr_1.1.4                
## [29] kableExtra_1.4.0            tidyr_1.3.1                
## [31] reshape2_1.4.4              ggplot2_3.4.4              
## 
## loaded via a namespace (and not attached):
##   [1] RColorBrewer_1.1-3      rstudioapi_0.15.0       jsonlite_1.8.8         
##   [4] magrittr_2.0.3          TH.data_1.1-2           farver_2.1.1           
##   [7] rmarkdown_2.25          zlibbioc_1.48.0         ragg_1.2.7             
##  [10] vctrs_0.6.5             memoise_2.0.1           RCurl_1.98-1.14        
##  [13] askpass_1.2.0           rstatix_0.7.2           htmltools_0.5.7        
##  [16] S4Arrays_1.2.0          plotrix_3.8-4           curl_5.2.0             
##  [19] broom_1.0.5             cellranger_1.1.0        SparseArray_1.2.3      
##  [22] plyr_1.8.9              sandwich_3.1-0          zoo_1.8-12             
##  [25] cachem_1.0.8            uuid_1.2-0              mime_0.12              
##  [28] lifecycle_1.0.4         pkgconfig_2.0.3         Matrix_1.6-5           
##  [31] R6_2.5.1                fastmap_1.1.1           
GenomeInfoDbData_1.2.11
##  [34] shiny_1.8.0             digest_0.6.34           colorspace_2.1-0       
##  [37] AnnotationDbi_1.64.1    confintr_1.0.2          textshaping_0.3.7      
##  [40] RSQLite_2.3.5           labeling_0.4.3          fansi_1.0.6            
##  [43] mgcv_1.9-1              httr_1.4.7              abind_1.4-5            
##  [46] compiler_4.3.1          bit64_4.0.5             fontquiver_0.2.1       
##  [49] withr_3.0.0             backports_1.4.1         BiocParallel_1.36.0    
##  [52] carData_3.0-5           DBI_1.2.1               ggstats_0.5.1          
##  [55] highr_0.10              quantreg_5.97           openssl_2.1.1          
##  [58] DelayedArray_0.28.0     equatags_0.2.0          gfonts_0.2.0           
##  [61] gtools_3.9.5            tools_4.3.1             zip_2.3.1              
##  [64] httpuv_1.6.14           glue_1.7.0              nlme_3.1-164           
##  [67] promises_1.2.1          grid_4.3.1              generics_0.1.3         
##  [70] xslt_1.4.4              gtable_0.3.4            tzdb_0.4.0             
##  [73] hms_1.1.3               katex_1.4.1             xml2_1.3.6             
##  [76] car_3.1-2               utf8_1.2.4              XVector_0.42.0
```

```
##  [79] ggrepel_0.9.5           pillar_1.9.0            stringr_1.5.1
##  [82] vroom_1.6.5             later_1.3.2             splines_4.3.1
##  [85] lattice_0.22-5          survival_3.5-7          bit_4.0.5
##  [88] SparseM_1.81            annotate_1.80.0         tidyselect_1.2.0
##  [91] fontLiberation_0.1.0    locfit_1.5-9.8          Biostrings_2.70.2
##  [94] knitr_1.45              fontBitstreamVera_0.1.1 V8_4.4.1
##  [97] svglite_2.1.3           crul_1.4.0              xfun_0.41
## [100] stringi_1.8.3           yaml_2.3.8              evaluate_0.23
## [103] codetools_0.2-19        httpcode_0.3.0          officer_0.6.3
## [106] gdtools_0.3.5           tibble_3.2.1            cli_3.6.2
## [109] xtable_1.8-4            systemfonts_1.0.5       munsell_0.5.0
## [112] Rcpp_1.0.12             XML_3.99-0.16.1         parallel_4.3.1
## [115] MatrixModels_0.5-3      ellipsis_0.3.2          blob_1.2.4
## [118] bitops_1.0-7            viridisLite_0.4.2       mvtnorm_1.2-4
## [121] scales_1.3.0            purrr_1.0.2             crayon_1.5.2
## [124] rlang_1.1.3             KEGGREST_1.42.0         multcomp_1.4-25
```
