# Supplementary material for: Effects of eicosapentaneoic acid on innate immune responses in an Atlantic salmon kidney cell line in vitro
Source: PLoS One. 2024 May 28;19(5):e0302286. doi: 10.1371/journal.pone.0302286 (PMC11132502; doi:10.1371/journal.pone.0302286)
Supplement: Attachment — Submitted filename: Response to reviewers PONE-D-23-38632.docx [file pone.0302286.s002.docx]

Response to reviewers PONE-D-23-38362

1. Please ensure that your manuscript meets PLOS ONE's style requirements, including those for file naming. The PLOS ONE style templates can be found at <https://journals.plos.org/plosone/s/file?id=wjVg/PLOSOne_formatting_sample_main_body.pdf> and <https://journals.plos.org/plosone/s/file?id=ba62/PLOSOne_formatting_sample_title_authors_affiliations.pdf>

**Response – the naming of figures, tables and supporting information is now in line with PONE style requirements**

2. We noticed you have some minor occurrence of overlapping text with the following previous publication(s), which needs to be addressed:

<https://link.springer.com/article/10.1007/s10695-017-0353-4>?

**Response: Yes, it is the description of the method for fatty acid analysis in the methods section. I have now inserted the citation to the article with the similar description (self citation). I could not find duplicated text outside the methods section.**

In your revision ensure you cite all your sources (including your own works), and quote or rephrase any duplicated text outside the methods section. Further consideration is dependent on these concerns being addressed."""

AAM (Straive) 28 Nov 2023: IRB for Animal Not Stated

To comply with PLOS ONE submissions requirements, please provide the following information in the Methods section of the manuscript and in the “Ethics Statement” field of the submission form (via “Edit Submission”):

* Please indicate whether an animal research ethics committee prospectively approved this research or granted a formal waiver of ethics approval.* Please enter the name of your Institutional Animal Care and Use Committee (IACUC) or other relevant ethics board. Also include an approval number if one was obtained.

* If anesthesia, euthanasia, or any kind of animal sacrifice is part of the study, please include briefly in your statement which substances and/or methods were applied.

For additional information about PLOS ONE submissions requirements for ethics oversight of animal work, please refer to <http://journals.plos.org/plosone/s/submission-guidelines#loc-animal-research>

**Response – this is a pure in vitro experiment using only an immortalized cell line (ASK) established many years ago. It is therefore not necessary with an approval from the Norwegian animal research ethics committee (FOTS) to conduct this study. I have stated this in the first section of the methods.**

3. Note from Emily Chenette, Editor in Chief of PLOS ONE, and Iain Hrynaszkiewicz, Director of Open Research Solutions at PLOS: Did you know that depositing data in a repository is associated with up to a 25% citation advantage (<https://doi.org/10.1371/journal.pone.0230416>)? If you’ve not already done so, consider depositing your raw data in a repository to ensure your work is read, appreciated and cited by the largest possible audience. You’ll also earn an Accessible Data icon on your published paper if you deposit your data in any participating repository (<https://plos.org/open-science/open-data/#accessible-data>).

**Response – the raw data from this study have already been deposited in the SRA archive (submission id: PRJNA1033415)**

4. We note that the grant information you provided in the ‘Funding Information’ and ‘Financial Disclosure’ sections do not match.

When you resubmit, please ensure that you provide the correct grant numbers for the awards you received for your study in the ‘Funding Information’ section.

**Response -**

5. Thank you for stating the following in the Acknowledgments Section of your manuscript:

" this study was supported via grant #901484 from the Norwegian Seafood Research fund (FHF). We thank Beata Mohebi, Truls Rasmussen and Målfrid Bjerke for skillful technical assistance."

We note that you have provided funding information that is not currently declared in your Funding Statement. However, funding information should not appear in the Acknowledgments section or other areas of your manuscript. We will only publish funding information present in the Funding Statement section of the online submission form.

Please remove any funding-related text from the manuscript and let us know how you would like to update your Funding Statement. Currently, your Funding Statement reads as follows:

"TG, BR and TKØ received grant #901484 from the Norwegian Seafood Research fund (FHF)

<https://www.fhf.no/>

The sponsor played no role in the experimental design"

Please include your amended statements within your cover letter; we will change the online submission form on your behalf.

**Response – I have removed funding info from the Acknowledgements section.**

6. Please ensure that you refer to Figure 8 in your text as, if accepted, production will need this reference to link the reader to the figure.

**Response: A reference to Fig 8 have now been included (around line 300)**

7. Please include captions for your Supporting Information files at the end of your manuscript, and update any in-text citations to match accordingly. Please see our Supporting Information guidelines for more information: <http://journals.plos.org/plosone/s/supporting-information>.

**Response: A caption for the supporting file have now been included at the end of the manuscript**

[Note: HTML markup is below. Please do not edit.]

Reviewers' comments:

Reviewer's Responses to Questions

Comments to the Author

1. Is the manuscript technically sound, and do the data support the conclusions?

The manuscript must describe a technically sound piece of scientific research with data that supports the conclusions. Experiments must have been conducted rigorously, with appropriate controls, replication, and sample sizes. The conclusions must be drawn appropriately based on the data presented.

Reviewer #1: Yes

Reviewer #2: Yes

________________________________

2. Has the statistical analysis been performed appropriately and rigorously?

Reviewer #1: Yes

Reviewer #2: Yes

________________________________

3. Have the authors made all data underlying the findings in their manuscript fully available?

The PLOS Data policy <<http://www.plosone.org/static/policies.action#sharing>> requires authors to make all data underlying the findings described in their manuscript fully available without restriction, with rare exception (please refer to the Data Availability Statement in the manuscript PDF file). The data should be provided as part of the manuscript or its supporting information, or deposited to a public repository. For example, in addition to summary statistics, the data points behind means, medians and variance measures should be available. If there are restrictions on publicly sharing data—e.g. participant privacy or use of data from a third party—those must be specified.

Reviewer #1: Yes

Reviewer #2: Yes

________________________________

4. Is the manuscript presented in an intelligible fashion and written in standard English?

PLOS ONE does not copyedit accepted manuscripts, so the language in submitted articles must be clear, correct, and unambiguous. Any typographical or grammatical errors should be corrected at revision, so please note any specific errors here.

Reviewer #1: Yes

Reviewer #2: Yes

________________________________

5. Review Comments to the Author

Please use the space provided to explain your answers to the questions above. You may also include additional comments for the author, including concerns about dual publication, research ethics, or publication ethics. (Please upload your review as an attachment if it exceeds 20,000 characters)

Reviewer #1: Summary

The article discusses the field of immunometabolism, focusing on the interaction between nutrients, energy metabolism, and the immune system in both health and disease. It emphasizes the limited understanding of the interplay between feed and immunity in fish, particularly in the context of dietary changes in Atlantic salmon. The traditional dietary sources of essential fatty acids, such as fish oil and fish meal, are being substituted by plant oils, impacting the levels of eicosapentaenoic acid (EPA) and docosahexaenoic acid (DHA) in salmon diets. The study explores the effects of different cellular levels of EPA on the transcriptional responses of salmon cells to the viral mimic poly I:C, revealing a potential link between EPA levels and the modulation of immune responses. The article is well-written, scientifically rigorous, and contributes valuable insights to the field of fish immunometabolism.

General Comments

I acknowledge the limitations of generalizing findings from Atlantic salmon cells to other fish species or different populations of Atlantic salmon. Explicitly discuss how the observed changes might impact the overall well-being and performance of fish.

**Response: we have now included a discussion of the relevance of in vitro studies for the understanding of fish health in the discussion with reference to new feeds and vaccines (around line 400)**

• The article primarily focuses on EPA, but it would be valuable to explore potential interactions with docosahexaenoic acid (DHA) since fish oil traditionally contains both EPA and DHA (Lines 68-69).

**Response: this is a good point and we have planned experiments where the role of DHA in innate immune responses against viral infections are studied**

• The reliance on in vitro cell culture experiments is noted. Consider addressing the limitations of the cell culture model and discussing how in vivo studies could complement these findings (Lines 110-112, 368-370).

**Response: This is an important point that not thoroughly discussed. We have therefore addressed these limitations in the Discussion (line 395-405) with additional references.**

• The study assesses short-term effects after one week. Consider discussing potential long-term effects or the sustainability of observed changes over extended periods (Lines 114-124).

**Response – this is the big question: translate from in vitro to in vivo. Although many of the effects of n-3 PUFA on immune cells observed in cell culture experiments can be recreated in vivo via feeding this is not always the case. We now have addressed this nin the introduction and included some important references on this matter.**

(1)

Recommendation

I recommend this manuscript for publication in Plos One. The study opens avenues for further research on the role of EPA in antiviral immunity in live fish and highlights the scarcity of similar studies in fish.

Reviewer #2: Dear authors,

Congratulations and you have done a good job. I have critically gone through the manuscript entitled “Effects of eicosapentaneoic acid on innate immune responses in an Atlantic salmon kidney cell line in vitro”. However, you need to incorporate the revision and answer the comments that are stated below before it is published.

1. Abbreviation should be expanded while writhing for the first time.

**Response – fixed**

2. L1 write the scientific name of Atlantic salmon.

**Response – fixed**

3. L28 and L30 write the full form of VEGF and PPAR.

**Response – fixed**

4. L42 write the full form of RLR signalling.

**Response - fixed**

5. Broken line analysis may use to know the exact EPA level rather than writing it as 200 uM EPA. It may be less or more than 200 uM EPA

**Response – this is a good suggestion and something we didn’t think about using in our analysis. We therefore tested the R package “segmented “ on some of our data to identify possible piecewise linear relations between EPA levels and transcriptional values. I think there are good reasons to believe that some of the responses are segmented. However our dataset only cover 5 concentrations of EPA, leaving too few datapoints to estimate 2 betas. This is something we will consider in the future in new experiments.**

________________________________

6. PLOS authors have the option to publish the peer review history of their article (what does this mean? <<https://journals.plos.org/plosone/s/editorial-and-peer-review-process#loc-peer-review-history>> ). If published, this will include your full peer review and any attached files.

If you choose “no”, your identity will remain anonymous but your review may still be made public.

Do you want your identity to be public for this peer review? For information about this choice, including consent withdrawal, please see our Privacy Policy <<https://www.plos.org/privacy-policy>> .

Reviewer #1: No

Reviewer #2: Yes: Gyandeep Gupta

________________________________

[NOTE: If reviewer comments were submitted as an attachment file, they will be attached to this email and accessible via the submission site. Please log into your account, locate the manuscript record, and check for the action link "View Attachments". If this link does not appear, there are no attachment files.]

While revising your submission, please upload your figure files to the Preflight Analysis and Conversion Engine (PACE) digital diagnostic tool, <https://pacev2.apexcovantage.com/>. PACE helps ensure that figures meet PLOS requirements. To use PACE, you must first register as a user. Registration is free. Then, login and navigate to the UPLOAD tab, where you will find detailed instructions on how to use the tool. If you encounter any issues or have any questions when using PACE, please email PLOS at [figures@plos.org](mailto:figures@plos.org) <<mailto:figures@plos.org>> . Please note that Supporting Information files do not need this step.

1.	Lorente-Cebrian S, Costa AG, Navas-Carretero S, Zabala M, Laiglesia LM, Martinez JA, Moreno-Aliaga MJ. An update on the role of omega-3 fatty acids on inflammatory and degenerative diseases. J Physiol Biochem. 2015;71(2):341-9.
